# Genome-wide Analysis of WD40 Protein Family in Human

**DOI:** 10.1038/srep39262

**Published:** 2016-12-19

**Authors:** Xu-Dong Zou, Xue-Jia Hu, Jing Ma, Tuan Li, Zhi-Qiang Ye, Yun-Dong Wu

**Affiliations:** 1 Lab of Computational Chemistry and Drug Design, Laboratory of Chemical Genomics, Peking University Shenzhen Graduate School, Shenzhen 518055, P. R. China; 2 College of Chemistry, Peking University, Beijing, 100871, P. R. China

## Abstract

The WD40 proteins, often acting as scaffolds to form functional complexes in fundamental cellular processes, are one of the largest families encoded by the eukaryotic genomes. Systematic studies of this family on genome scale are highly required for understanding their detailed functions, but are currently lacking in the animal lineage. Here we present a comprehensive *in silico* study of the human WD40 family. We have identified 262 non-redundant WD40 proteins, and grouped them into 21 classes according to their domain architectures. Among them, 11 animal-specific domain architectures have been recognized. Sequence alignment indicates the complicated duplication and recombination events in the evolution of this family. Through further phylogenetic analysis, we have revealed that the WD40 family underwent more expansion than the overall average in the evolutionary early stage, and the early emerged WD40 proteins are prone to domain architectures with fundamental cellular roles and more interactions. While most widely and highly expressed human WD40 genes originated early, the tissue-specific ones often have late origin. These results provide a landscape of the human WD40 family concerning their classification, evolution, and expression, serving as a valuable complement to the previous studies in the plant lineage.

The WD40 domains, as special cases of the β-propeller domains, are abundant in eukaryotic proteomes. It was estimated that WD40 domain-containing proteins (WD40 protein family) account for about 1% of the human proteome^1^. A canonical WD40 domain comprises 7 blades or repeats, each of which contains 40–60 residues with a motif of WD (tryptophan and aspartic acid). The blades then fold into a propeller, exposing the top, bottom, and side surfaces, which are believed to be involved in molecular recognition and interaction^2^.

The WD40 domains often act as scaffolds to recruit other molecules, forming functional complexes or protein-protein interactions^3^,^4^,^5^. WD40 proteins play important roles in many fundamental biological processes such as signal transduction^6^, histone modification^7^, DNA damage response^8^, transcription regulation^9^,^10^, RNA processing^11^, protein degradation^12^, and apoptosis^13^. Consistent with their essential roles, many are involved in various diseases. For example, FBXW7 is a well-known tumour suppressor and is implicated in several cancers^14^,^15^. TLE1 is also a well-studied tumour suppressor gene^16^. Besides tumour, other diseases are involved in as well: WDR45 is associated with neurodegeneration through autophagy^17^, and WDR62 was found mutated in human microcephaly^18^.

Concerning their important roles in basic biological processes and their abundance, it is valuable to perform a genome-wide computational analysis on this family of proteins. Currently, several genome-wide studies have already put efforts on identifying and analysing WD40 protein family in plants including Arabidopsis, rice, foxtail millet, and cucumber^19^,^20^,^21^,^22^. These studies found variation in the number of WD40 genes in different plants, suggesting gene expansion history during evolution. While all of these studies speculated that most WD40 genes in plants are conserved across all the eukaryotes, they are functionally diverse between the family members. In rice and foxtail millet, the authors classified WD40 proteins into 11 and 12 classes based on their domain architectures, respectively^20^,^22^. Evolutionary analysis showed that both tandem duplication and segmental duplication contributed to the expansion of WD40 gene family, and revealed that plant-specific domain architectures and functions emerged with the family expansion in the plant lineage. In a study of tomato genome, the authors specifically analysed the DDB1-binding WD40 proteins, a subfamily presumably serving as substrates recognition components of CUL4 E3 ligases, and experimentally confirmed 14 proteins interacting with DDB1^23^.

This kind of studies provided us a global landscape of the characteristics of WD40 family, including classification, evolution, expression, and functions in plants. However, the systematic study of WD40 protein family in the animal lineage is lacking. Since the plant kingdom has undergone genome evolution significantly different from the animal kingdom after their divergence, the genome-wide analysis of the WD40 protein family in animals should result in novel insights other than those from plants research, and will thus serve as a complement of a more comprehensive landscape.

In this work, we chose human as a representative of the animal lineage for a genome-wide computational analysis. First, a reliable set of human WD40 proteins were identified carefully. Second, we roughly depicted their domain architectures and made a classification followed by inspecting the functional annotations. Detailed sequence comparison at the level of domain and repeat was further performed. Third, their phylogenetic relationships and evolutionary implications were proposed. Fourth, WD40 genes with different expression profiles and their relationship with phylogenetic patterns were studied as well. This analysis provided a broad understanding of the WD40 protein family in the animal lineage, and offered a good basis for further investigation of biological functions and evolution of animal WD40 proteins. More specifically, the study on human WD40 proteins will hopefully provide crucial clues in the research of diseases and health.

## Results

### 262 non-redundant human WD40 proteins are identified

We utilized the WDSP tool to identify WD40 proteins in human reference proteome^24^,^25^. The careful curation pipeline (Supplementary Fig. S1) resulted in 262 non-redundant human WD40 (*hs*WD40) proteins, each of which represents the typical protein product of an *hs*WD40 gene (Table 1, Supplementary Table S1). In brief, these *hs*WD40 proteins contain more than 300 WD40 domains, which are composed of 2188 WD40 repeats. Among them, 167 out of 262 (63.7%) *hs*WD40 proteins hold exactly 7 repeats, indicating that more than half of the proteins should contain the canonical form of WD40 domains. In addition, a small part of *hs*WD40 proteins are composed of more than 10 repeats, suggesting the existence of multiple WD40 domains within the same protein. In extreme cases, some *hs*WD40 proteins even contain more than 20 repeats, such as WDR6, EML5, and EML6, which contains 20, 33, and 35 repeats, respectively.

### Domain architectures can define 21 classes of *hs*WD40s

It is known that many WD40 proteins may contain other types of domains to form complicated domain architectures, and this may endow WD40 family with complicated functions. To obtain the panorama, we annotated the domain architectures of the *hs*WD40 proteins, and inspected their functions from literature subsequently.

**Figure 1:**
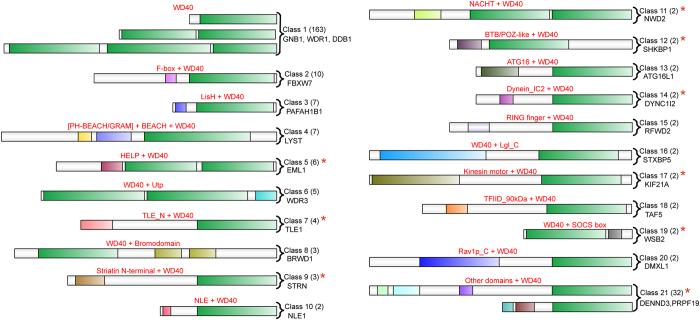
Domain architectures of *hs*WD40 proteins. The schematic domain architectures of representative *hs*WD40 proteins were roughly depicted. The WD40 domains are coloured in green, and other domain types are filled in other different colours separately. Red texts describe the classes of domain architectures. The number of members in each class is given in the parentheses with the name of the representative protein shown below. Red stars indicate the potential animal-specific domain architectures.

Based on their domain architectures, we grouped *hs*WD40s into 21 classes (Fig. 1). One hundred and sixty-three *hs*WD40 proteins containing only WD40 domains were grouped into Class 1, and the rest 99 *hs*WD40 proteins containing additional domains were grouped into classes from 2 to 21. For example, 10 *hs*WD40s with F-box and WD40 domain were classified as Class 2, and 7 *hs*WD40 proteins in Class 3 contain LisH domain. For the sake of simplicity, we put all domain architectures with only one member into Class 21 (details in Supplementary Table S1). The grouping of domain architectures provided crucial information concerning their subfamily classification. Inspecting their functional information from the literature revealed that *hs*WD40 proteins with the same domain architecture generally function in a similar way or are involved in similar functional modules (Supplementary Table S2).

When comparing between human and plants (Arabidopsis, rice, foxtail millet, and cucumber), we found that many domain architectures are conserved. In detail, Class 2 (F-box + WD40), Class 3 (LisH + WD40), Class 4 (BEACH + WD40), Class 6 (WD40 + Utp), Class 8 (WD40 + Bromodomain), Class 10 (NLE + WD40), Class 13 (ATG16 + WD40), Class 15 (RING finger + WD40), Class 16 (WD40 + Lgl_C), and Class 18 (TFIID_90 kDa + WD40) are present in human and at least one plant species^20^,^22^.

In addition to these conserved domain architectures, we also noticed 11 potential animal-specific ones (marked with red stars in Fig. 1). They are Class 5 (HELP + WD40), Class 7 (TLE_N + WD40), Class 9 (Striatin N-terminal + WD40), Class 11 (NACHT + WD40), Class 12 (BTB/POZ-like + WD40), Class 14 (Dynein_IC2 + WD40), Class 17 (Kinesin motor + WD40), Class 19 (WD40 + SOCS box), and at least another three architectures in Class 21 (WD40 + U box + SAM, WD40 + RWD, CARD + NB-ARC + WD40), none of which was reported in the previous plants studies. Proteins with these domain architectures may have specifically emerged in the animal lineage, and it is reasonable to speculate that they may carry out animal-specific functions. To confirm this, we performed functional enrichment analysis for proteins belonging to these architectures, and identified six significantly enriched Gene Ontology (GO) biological processes (*p*-value < 0.05), including β-catenin-TCF complex assembly, Wnt signalling pathway, microtubule-based movement, microtubule cytoskeleton organization, animal organ morphogenesis, and protein homooligomerization (Supplementary Table S3). Among them, both β-catenin TCF complex and Wnt signaling are important for embryonic development in animals rather than in plants. The two microtubule-related processes in animals were reported to be distinct from those in plants. The fifth enriched GO term, as its name implies, is apparently animal-specific. These results are well consistent with our speculation.

### Sequence alignment suggested further subfamily classification, and duplication after recombination events

For such a large family, it is necessary to investigate their relationships with each other and the subfamily classifications. The domain architectures, serving as a kind of rough sequence feature, were analysed and grouped in the previous section. In order to obtain the more detailed relationships among *hs*WD40 family members, we explored the pairwise alignments of domain sequences.

**Figure 2:**
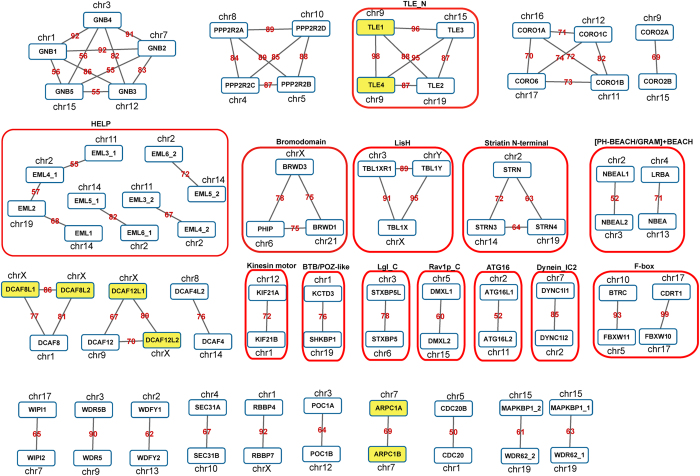
Clusters of highly similar *hs*WD40 domains. The nodes represent *hs*WD40 domains, and the edges indicate that the sequence similarities between them are high. The texts in the nodes are the gene symbols, and the numbers on the edges show the domain sequence identities in percentage. If multiple WD40 domains come from the same protein, a numeric suffix is added to avoid confusion. Domains from tandemly arrayed genes (TAGs) are yellow-shaded, and the red boxes indicate the same domain architecture classes for multi-domain WD40 proteins.

Although the WD40 domain sequences are very diverse in general^1^, we identified 71 pairs (0.16% of all pairwise comparisons, 86 different domain sequences involved) of highly similar domains, and a considerable number of them are connected into clusters (Fig. 2, Supplementary Table S4), suggesting that members in the same cluster can be classified into a subfamily reasonably. For example, the first 2 clusters in Fig. 2, although both of which belong to Class 1 according to the domain architecture, further defined the subfamilies of GNB and PPP2R2 respectively. Many other clusters also meet this scenario, so the WD40 domain sequence alignment indeed provided more details concerning the subfamily classification.

It is well known that gene families should have evolved by complicated gene duplication events^26^. Since the sequence divergences within each cluster are less than those between clusters, the domains within each cluster in Fig. 2 may have evolved by duplication events more recent than those between clusters. As for proteins with multiple domain types, it is accepted that domain recombination events also happened in the evolution in addition to the duplication^27^. It will deepen our understanding to discriminate the earlier events from the later ones. Interestingly, we noticed that there exists evident consistency between WD40 domain sequence similarities and the overall domain architectures of the proteins. That is, when the WD40 domain sequence similarity of two proteins is high (connected in Fig. 2), the two proteins almost always belong to the same class of domain architecture (Fig. 2, rounded rectangles). For example, the WD40 domain sequences of TLE1-4 are highly similar between each other, and all of them belong to Class 7 (TLE_N + WD40), and so do BRWD1, BRWD3, and PHIP, which belong to Class 8 (WD40 + Bromodomain). These results suggested that the whole gene duplication events happened pervasively after the domain recombination in the evolution history of the multi-domain WD40 proteins. If it was not the case, we should have detected highly similar domain pairs coming from different domain architecture classes.

Since each WD40 domain contains multiple repeats, we further performed the pairwise sequence alignment at the repeat level, and found 596 pairs of highly similar repeats (0.025% of all pairwise comparisons, including 655 different repeats, covering 121 different proteins). More than 75% of highly similar repeat pairs came from highly similar domain pairs. Moreover, we noticed that 7 pairs of highly similar repeats came from within-domain repeat alignment, *i.e.*, FBXW7, DAW1, and WDR5 (Supplementary Table S5). These data suggested that WD40 domain also evolved at the repeat-level through recent repeat duplication in addition to the domain-level duplication, although the latter should be the dominant^28^.

### *hs*WD40 genes are widely distributed on all chromosomes

It will provide us an overall picture and more evolutionary implications to sketch a “WD40 map” by plotting all the *hs*WD40 genes according to their chromosomal locations. We thus extracted their chromosomal coordinates from Ensembl web site and made a circular map (Supplementary Fig. S2). Overall, the “WD40 map” can offer us a brief landscape for quickly browsing their genomic locations and contexts. As shown in the “WD40 map”, *hs*WD40 genes are widely distributed on all chromosomes. With the number of protein-coding genes on each chromosome as a denominator, the percentage of *hs*WD40 genes ranges from 0.36% on chromosome 20 to 2.11% on chromosome 9 (Supplementary Table S6). Overall, the number of *hs*WD40 genes on each chromosome is roughly proportional to that of all protein-coding genes on it, though several evidently biased cases exist. Specifically, the percentages of *hs*WD40 genes in chromosome 9, 3, and 2 are 2.11%, 2.04%, and 1.87%, respectively, which are significantly higher than the overall average, *i.e.*, 1.29% (*p*-values: 0.032, 0.023, and 0.043, respectively). On the contrary, the percentages of *hs*WD40 genes in chromosome 20 and 11 are 0.36% and 0.76%, respectively, which are significantly lower than the overall average (*p*-values: 0.023 and 0.043).

Genome segmental duplication and tandem duplication play important roles in the evolution of a gene family^29^,^30^. The genomic locations and pairwise sequence similarities illustrated in the “WD40 map”, revealed that pervasive segmental duplication events have acted in the expansion history of the WD40 gene family. In addition, we identified 4 pairs of tandemly arrayed genes (TAGs), *i.e.*, TLE1 and TLE4, DCAF8L1 and DCAF8L2, DCAF12L1 and DCAF12L2, and ARPC1A and ARPC1B. These TAGs should have been involved in tandem duplication events (red gene symbols in Supplementary Fig. S2, and yellow shading in Fig. 2).

### WD40 family underwent more expansion than overall average in evolutionary early stage, but less in late stage

The analyses in previous sections glimpsed several evolutionary perspectives of *hs*WD40s, and more insights will be disclosed if we further study them in the context of an evolutionary tree with pivotal time points, as different members of the *hs*WD40 protein family should have emerged at different evolutionary stages, and may thus be implicated in different functions.

**Figure 3:**
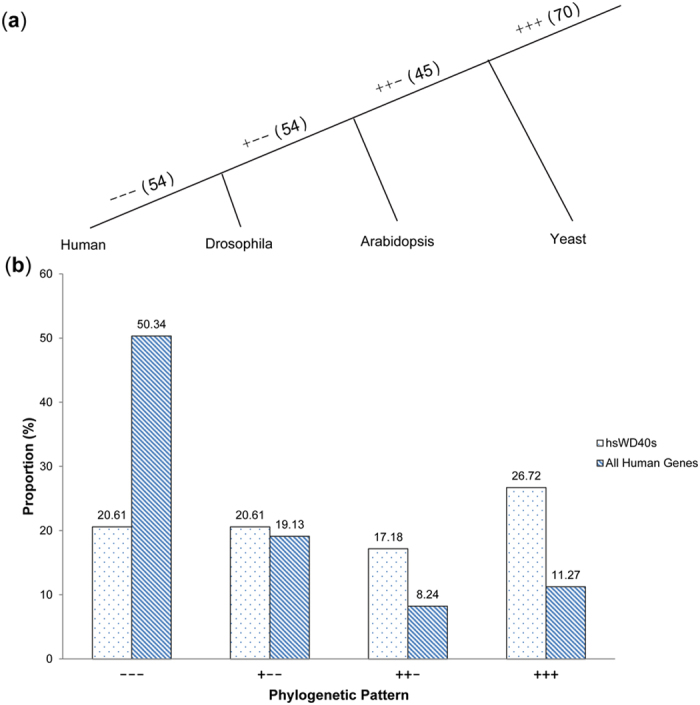
The proportions of *hs*WD40 genes with different phylogenetic patterns. The phylogenetic patterns of human WD40 genes and all human protein-coding genes (background genes) were compared. (**a**) Phylogenetic relationships of the 4 representative species, *i.e.*, human, Drosophila, Arabidopsis, and yeast. The symbols of “+++”, “++−”, “+−−”, and “−−−” denote the different phylogenetic patterns (Methods). The numbers in parentheses give the counts of *hs*WD40 genes accordingly. (**b**) Comparison between the proportions of *hs*WD40 genes and all human protein-coding genes in different phylogenetic patterns. Bars filled with dots and slashes represent the percentages of *hs*WD40 genes and those of all human protein-coding genes, respectively.

We performed a phylogenetic analysis roughly according to their status of ortholog existence (referred to as phylogenetic pattern) in three model organisms, *i.e.*, yeast, Arabidopsis, and Drosophila. These organisms, in addition to human, are representatives for single-cell eukaryotes, plants, invertebrates, and vertebrates, whose speciation events can define several key time points in the evolutionary tree. The human genes with orthologs in all other three species (70 in total, labelled as ‘+++’ in Fig. 3) indicate their emergence may be as ancient as the origin of eukaryotes. Besides these 70 *hs*WD40s, there are 45 *hs*WD40 genes with orthologs only in Arabidopsis and Drosophila (labelled as ‘++−’), suggesting that these WD40s might have emerged before the separation of plants and animals. And 54 *hs*WD40 genes have orthologs only in Drosophila (labelled as ‘+−−’), indicating that they might have emerged before the separation of invertebrates and vertebrates. Another 54 *hs*WD40 genes without orthologs in any of the other 3 species should have originated after the separation of vertebrates from invertebrates (labelled as ‘−−−’).

When comparing *hs*WD40s with all human protein-coding genes, we can infer that a larger proportion of *hs*WD40s than that of all genes (26.72% *vs*. 11.27%) should have originated at the very early stage of eukaryotes (Fig. 3(a,b), Supplementary Tables S7 and S8). A similar speculation can be deduced for the genes originated before the separation of animals and plants (17.18% *vs*. 8.24%). However, there is no such tendency before the separation of invertebrates and vertebrates (20.61% *vs*. 19.13%). Furthermore, this kind of tendency is inverted after the separation of vertebrates from invertebrates (20.61% *vs*. 50.34%, Fig. 3(a,b), Supplementary Tables S7 and S8).

Studies of human genome showed that many human gene families have largely expanded in the late stage of evolution^31^. Distinct from this, our observations indicated that the WD40 family has undergone more expansion than the overall average of all genes during the early evolutionary period, which echoes the fundamental cellular functions (*i.e.*, house-keeping) enriched in WD40 genes.

On the other hand, the WD40 family underwent less expansion than the overall average after the separation of vertebrates and invertebrates. Though they expanded less, the *hs*WD40s with animal or vertebrate origin may have evolved some animal or vertebrate-specific functions other than fundamental ones. Further studies on these *hs*WD40s may lead to discoveries concerning their important biological roles. For example, AHI1, as one of them, has been demonstrated that its mutations can result in JBTS, a human disease characterized by psychomotor delay, cerebellar hypoplasia, consecutive ataxia, and so on^32^.

### Different phylogenetic patterns are associated with different domain architectures and interaction counts

**Figure 4:**
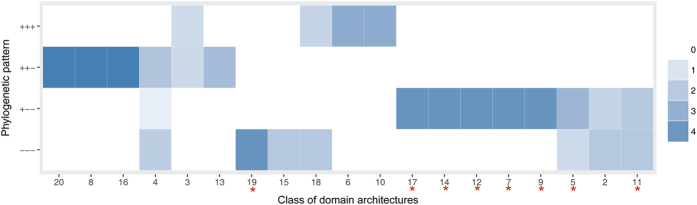
Domain architectures in *hs*WD40 proteins with four different phylogenetic patterns. The numbers at the horizontal axis denote the classes of domain architectures, and the vertical axis gives out the four phylogenetic patterns with different time of evolutionary origin. The colour gradient in each cell represents the relative count of proteins matching the two axes, which were adopted to sort the columns through clustering. Red stars at the horizontal axis indicate the potential animal-specific domain architecture classes.

Both phylogenetic patterns and domain architectures can be utilized for functional inference, so we further inspected the domain architectures with different phylogenetic patterns (Fig. 4). We found that domain architectures of Class 3, 6, 10, and 18 emerged at the early stage of eukaryotes (phylogenetic pattern of “+++”). According to their functional annotations (Supplementary Table S2), proteins in these classes are involved in very fundamental functions such as transcription regulation, histone binding, and rRNA processing. WD40 proteins that emerged at multi-cellular stage (phylogenetic pattern of “++−”) began to present domain architectures of Class 4, 8, 13, 16, and 20, which endowed proteins with functions of apoptosis, autophagy, cell morphology, and neurotransmitter release process. After the divergence of plant and animal (phylogenetic pattern of “+−−”), more domain architectures emerged, including Class 2, 5, 7, 9, 11, 12, 14, and 17. Among them, Class 5, 14, and 17 are related to microtubule dynamic processes which is different between animal and plant, and Class 9 and 11 are implicated in estrogen or androgen receptor binding. The WD40 proteins that emerged after the separation of vertebrate from invertebrate (phylogenetic pattern of “−−−”), are composed of domain architectures of Class 15, 19, and several other architectures that had already emerged at earlier stages. Class 15 and 19 in this group, and Class 2 and 12 in the group of “+−−”, are implicated in E3 ubiquitin ligase system, which may be corresponded to that the degradation system in organisms with more complicated cellular structures need to recognize more protein substrates. It is worth noting that almost all of the potential animal-specific domain architectures consistently belong to the phylogenetic group of “+−−” or “−−−” (marked with red stars in Fig. 4), which meets our expectation very well.

Since WD40 proteins are frequently involved in protein-protein interactions (PPI), we further briefly checked their network degrees in a curated human PPI dataset^33^, where 174 of the 262 WD40 proteins have interaction data. In this dataset, about 55% of the interactions involved in multi-domain WD40 proteins should be contributed from WD40 domains according to the estimation of a domain-domain prediction method^34^. Although there is no evident trend for the degrees of the four groups with different phylogenetic patterns, the degrees of WD40 proteins in group “+++” is significantly higher than those in group “−−−” with a fold change of ~2.75 (18.54 *vs*. 6.74, *p*-value = 0.93e-3). This indicates that the late emerged proteins should be involved in fewer interactions than the early ones, possibly because they have undergone shorter evolutionary time.

### Most widely and highly expressed *hs*WD40 genes originated early in evolution, while most tissue-specific ones have late origin

Compared to the static features including domain architectures, sequence similarities, genomic locations, and phylogenetic properties, the gene expression profile across various tissues further presents a more vivid picture concerning the biological activity and functions of a gene. To view the expression patterns of the 262 *hs*WD40 genes, we used the RNA-seq dataset from the Human Protein Atlas, which contains normalized gene expression levels across 27 tissues^35^ (Supplementary Table S9). According to the expression profiles, all *hs*WD40 genes have detectable expression signals in at least one tissue. Overall, the median expression levels of WD40 genes are two times higher than those of all human genes in all tissues (Supplementary Fig. S3). Since a considerable portion of *hs*WD40 genes may originate at the evolutionarily early stage of eukaryotes and play roles in basic cellular processes (or “house-keeping” in other words), it is reasonable to witness their overall higher expression levels.

In addition to the overall expression pattern, the *hs*WD40 genes can be further divided into several classes according to their differentiated expression profiles. According to our definition in Methods, 204 *hs*WD40 genes can be classified as “Expressed in all”, *i.e.*, most of *hs*WD40 genes are widely expressed. Furthermore, among them, 52 can be grouped as “Highly expressed in all”, implying that the functions of these genes should be enriched with house-keeping roles in fundamental cellular processes (Supplementary Table S9).

Except the widely expressed genes, we also identified 20 *hs*WD40 genes which manifested the “Tissue-specific” expression characteristics (Table 2, Supplementary Table S9). Among them, 17 genes are specifically expressed in testis, while the other 3 are specifically expressed in brain, prostate, and pancreas, respectively. This small list of the *hs*WD40 genes may have evolved with specific functions rather than house-keeping.

**Figure 5:**
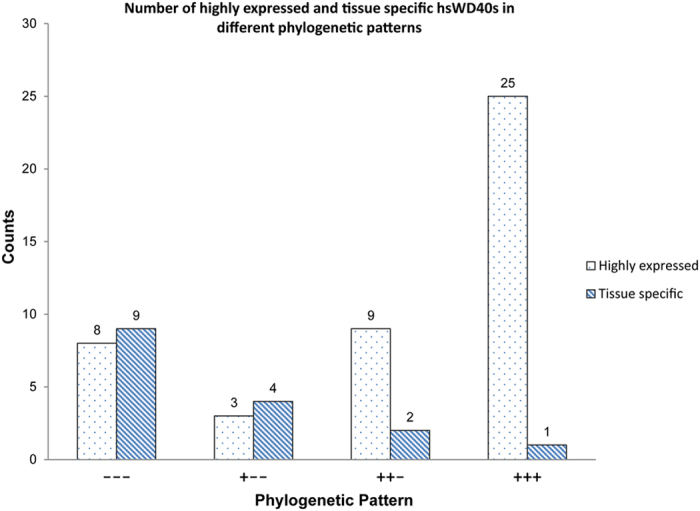
Comparison between the numbers of “Highly expressed in all” and “Tissue-specific” *hs*WD40 genes in different phylogenetic patterns. The bars filled with dots and slashes represent the counts of “Highly expressed in all” and those of “Tissue-specific” *hs*WD40 genes, respectively.

Both the expression profile and phylogenetic information can give us indications about the functions of genes, so integrating them together may present some interesting patterns and provide deeper insights. Bearing this in mind, we combined the classification of expression and the phylogenetic pattern of *hs*WD40 genes, and found that the “Highly expressed in all” WD40 genes and the “Tissue-specific” genes showed strikingly different distribution of phylogenetic patterns (Fig. 5). The WD40 genes that expressed highly in all tissues reside dominantly in the phylogenetic group with very early evolutionary origin (labelled as ‘+++’ in Fig. 5) among all the four representative groups. Since the *hs*WD40 genes with very ancient origin were supposed to play fundamental roles in basic cellular processes according to the previous section, and so were the *hs*WD40s with wide and high expression (*i.e.*, house-keeping) according to this section, it is reasonable to observe this coincidence. In contrast, the WD40 genes whose expressions are tissue-specific fall dominantly into the phylogenetic group with late evolutionary origin (labelled as ‘−−−’ in Fig. 5, *i.e.*, originated after the separation of vertebrates and invertebrates). We have speculated that *hs*WD40 genes with late evolutionary origin may have evolved with lineage-specific functions, and here the tissue-specific expression patterns actually serve as certain kind of evidences since specialized tissues or organs only occurred in specific lineage. Overall, analysing the *hs*WD40 family with both the dimensions of phylogeny and expression can provide us deeper insights, and can further help researchers choose individual WD40 genes for detailed functional studies with experiments.

## Discussion

Due to the low sequence similarity between WD40 repeats, and the variable number of repeats within a single WD40 domain, it is a big challenge to identify WD40 domains by methods merely based on sequence similarity search and alignment. In this work, we utilized the WDSP^24^ software, a tool designed for annotating WD40 repeats and domains specifically, to identify human WD40 domains. Rather than general methods which can only find typical WD40 repeats, WDSP is capable of detecting non-typical repeats with remote homology. Steven van Nocker defined a protein with 4 or more WD40 repeats to be a WD40 domain^19^, but we found that it should be at least 6 repeats to form a complete WD40 β-propeller according to the current 3D structures in PDB database. Hence, we defined a protein with six or more WD40 repeats to be a WD40 protein in this work, which should be more reliable.

In the classification based on the domain architectures, the 262 *hs*WD40 proteins were grouped roughly into 21 classes. It is worth noting that proteins in Class 1 are different from each other in sequence lengths, repeat numbers, and many other features. More efforts are required to make further classifications, and the domain sequence alignment that followed demonstrated its necessity. In addition, the Class 21 contains many different domain architectures with only one member identified, so it can actually be divided into many smaller groups. According to our domain annotation criteria, F-box domain was not identified in CDRT1, but annotations in some other databases with loose criteria did. This means that the domain architecture classification can be refined with more comprehensive domain annotations. We identified the potential animal-specific domain architectures by checking the literatures of plant studies, which may be improved by a more comprehensive comparative genomics study.

In the domain sequence alignment, it is not self-evident to define the WD40 domain boundaries of the proteins with multiple WD40 domains. Although we have considered this problem carefully according to our experiences, it will be improved if more accurate solutions of domain boundary definition are available. In the sequence comparison, we set 50% of sequence identity as the cut-off. This is a strict measure of sequence similarity, so we only considered the similar pairs of domains or repeats with high confidence. In this setting, 214 out of 300 domains were isolated with no similarity to other domains. If we lower the cut-off, more sequence pairs can be identified.

The pervasive but uneven distribution of *hs*WD40 genes on chromosomes is similar to those in plants^20^,^21^,^22^, which may be correlated with different levels of segmental duplication on different chromosomes. For example, the high density of *hs*WD40 genes in chromosome 9 may be related to the enriched segmental duplications^36^, but further elucidation of these distribution patterns needs more detailed investigations.

Among those *hs*WD40s with orthologs in other species, we noticed that some different human genes are co-orthologous to only one gene in other species (Supplementary Table S7), indicating a specific type of gene expansion. For instance, there are 5 human Gβ genes (GNB1–5), which are all orthologous to the same gene in Arabidopsis (GB1) and yeast (STE4). Another case is protein phosphatase 2, subunit B. There are 4 genes in this group (PPP2R2A, PPP2R2B, PPP2R2C, PPP2R2D), which are all orthologous to the same gene in Drosophila (tws) and yeast (CDC55). As expected, the 5 Gβ genes and the 4 phosphatase genes were all involved in the aforementioned highly similar domain clusters (Fig. 2). Although these phylogenetic data may reflect expansion within cluster happened in the evolutionary late stage, the fact that these genes have orthologs in all other species indicated that their “prototype” originated very early.

Gene expression profiles in 27 normal tissues were used in this study for mining functional implications. There are many gene expression datasets in the public domain with different levels of quality. Further mining of these data with careful curation and robust algorithms in the future will greatly improve our understanding of this gene family, especially for those datasets with disease and normal tissue comparisons. Previous studies have reported many WD40 genes involved in different human diseases, such as cancer and neurodegenerative diseases^14^,^15^,^17^,^37^,^38^,^39^. Studies on their biological roles in disease pathogenesis will be an important direction in the future.

Due to the unique structural features of the WD40 family, further systematic studies of them may be conducted, such as the hydrogen bond network, protein-protein interaction hotspots, and so on, from the perspective of evolution and subfamily classification. In addition, the analysis of these structure features may also be adopted to interpret or to help discriminate the disease-related mutations on WD40 domains, since the next-generation sequencing technology are identifying more and more variants by re-sequencing different samples.

## Conclusion

In this work, we presented a comprehensive characterization of the human WD40 protein family. 262 *hs*WD40 genes have been identified, and classified into 21 classes based on their domain architectures. Many architecture types were not observed in plants, and may be animal-specific. The domain sequence alignment provided detailed information regarding further subfamily classification, and indicated duplication and recombination events in evolution. The WD40 family should have undergone more expansion than overall average in the evolutionarily early stage, but experienced less expansion in the late stage. The early emerged WD40 proteins generally interact with more other proteins, and carry domain architectures playing roles in fundamental cellular processes. As for the gene expression, the overall transcription levels of WD40 genes are much higher than those of all human genes. Fifty-two *hs*WD40 genes are highly expressed in a wide spectrum of tissues, while 20 *hs*WD40 genes are tissue-specific. After integration of the phylogenetic patterns and expression profiles, we found that most widely and highly expressed *hs*WD40 genes originated early in evolution, while most tissue-specific ones have late origin.

Our work depicted a landscape of the *hs*WD40 protein family, including the subfamily classification, evolution, and gene expression. As the first systematic study of animal WD40 protein family, it can serve as an important complement to the published studies in plants, and do have identified animal-specific WD40s. These analyses provided crucial insights regarding their evolutionary and functional implications, and will thus help us prioritize important ones for further experimental investigations.

## Methods

### Identification of WD40 proteins from the human proteome

The sequences of human reference proteome were downloaded from UniProt in April 2014 (http://www.uniprot.org/proteomes/, UP000005640, UniProt Release 2014_03)^25^. WDSP software was adopted in a strict pipeline for the identification of human WD40 proteins (Supplementary Fig. S1). In brief, WDSP predicts out the potential repeats and calculates an average score for them. According to the previous experiences^24^, the minimum number of repeats is 6 for WD40 domains with known structures, and the repeat score of WDSP as high as 48 will greatly reduce the false positive predictions. So we set 48 as the cut-off of the average score and 6 as the cut-off of the number of repeats to screen the potentially reliable human WD40 proteins. The proteins that passed the filter were mapped to Ensembl gene identifiers and gene symbols by BioMart (http://grch37.ensembl.org/index.html, Genome assembly version GRCh37.p13)^40^, and only the longest sequence was kept if multiple proteins were mapped to the same gene. Through manual curation, a protein was discarded if there exist clear annotations denoting that it should belong to other β-propeller proteins. This procedure ensured that the final WD40 protein set is reliable and non-redundant.

### Determination of domain architectures

Domain annotation of WD40 proteins was performed locally using InterProScan 5 (version 5.10−50.0)^41^, with three domain annotation engines enabled, including ProDom-2006.1, SMART-6.2, and PfamA-27.0. The WD40 repeats annotated by InterProScan were replaced with annotations by WDSP, since WDSP can provide more complete and precise WD40 repeat annotations^24^. Based on the domain annotations, proteins with similar domain architectures were assigned to the same class. Schematic diagram for the domain architectures of *hs*WD40 proteins was drawn by using IBS^42^. The functional enrichment was analysed by using DAVID^43^,^44^ online, and the GO functional terms with *p*-values less than 0.05 were considered as enriched significantly.

### Domain and repeat sequence alignment

Pairwise sequence alignment for WD40 domains and repeats were performed by the BLASTP program with default parameters^45^. A protein may contain multiple WD40 domains. For the sake of simplicity, we split them sequentially by seven repeats, and every seven repeats were regarded as an individual WD40 domain. If six repeats were left, we also consider them as an individual domain, and if less than six repeats were left, they were discarded in this analysis. If a protein contains multiple WD40 domains, each domain were named after the gene symbol (or the protein ID) and a numeric suffix to avoid confusion. In the repeat alignment, we named each repeat based on the protein ID and a numeric suffix. If two sequences in an alignment resulted in identity greater than 50%, and the average coverage of the two sequences in the aligned region was greater than 90%, they were defined as a highly similar sequence pair. The graph of highly similar WD40 domain sequence pairs was prepared using Cytoscape^46^, and manual editing was added for more detailed information such as the chromosome numbering and additional domain names.

### Chromosomal localization

Coordinates of *hs*WD40 genes in human genome were obtained from Ensembl website through BioMart (http://grch37.ensembl.org/index.html, Genome assembly version GRCh37.p13)^40^. As BOP1 (ENSG00000261236) and CIRH1A (ENSG0000262788) do not locate on well-assembled chromosomes, only 260 genes were involved in the “WD40 map”, which was built using Circos^47^. The hyper-geometric distribution test was used to detect the chromosomes with biased WD40 abundance. Among the WD40 genes with highly similar domains, we also defined two WD40 genes adjacent to each other on the same chromosome with at most one spacer gene as tandemly arrayed genes (TAGs)^48^.

### Phylogenetic analysis and PPI network study

Orthologs of human genes in Drosophila, Arabidopsis, and yeast were obtained from InParanoid 8 (http://inparanoid.sbc.su.se/, Version 8.0)^49^. According to the status of ortholog existence, the genes were classified into different phylogenetic patterns. Specifically, the status of orthologs existence in “Drosophila, Arabidopsis and yeast”, “only Drosophila and Arabidopsis”, “only Drosophila”, and “none of the other three species”, are represented by the symbols of “+++”, “++−”, “+−−”, and “−−−”, respectively. The different phylogenetic patterns can be used to indicate the different time of evolutionary origin.

The observed number of proteins was counted for each combination of the domain architecture classes and the phylogenetic patterns, and the expected number for each combination was calculated with the assumption of independent marginal distributions. The ratios of observation to the expectation, *i.e.*, a kind of measure of relative counts of proteins matching specific domain architecture types and phylogenetic patterns, were subjected to hierarchical clustering (Euclidean distance and average linkage) for putting together domain architectures with similar distributions in phylogenetic patterns. The figure was prepared in the R programing environment, and the colour depth represents the ratio. Class 1 and 21 were not presented, since Class 1 contains proteins with only WD40 domains and Class 21 actually contain many kinds of domain architectures.

The human PPI dataset were downloaded from HIPPIE^33^ (v2.0), and only the PPIs detected by at least two methods and with a score of at least 0.5 were used for further analysis. Degree of each node in the PPI network was calculated by using Cytoscape^46^, and the Wilcoxon rank-sum test was performed to test whether there are significant differences between the degrees of WD40 proteins with different phylogenetic patterns. Domain-domain interaction prediction was performed by a parsimony approach implemented by linear programming^34^. Because the large amount of PPIs (more than 70,000) in the dataset impeded a thorough computation, we randomly sampled 2,000 PPIs for predicting the DDIs, and repeated the process for 1,000 times. For each run, we calculated the percentage of WD40 domain-mediated PPIs in multi-domain WD40 protein-associated PPIs, to estimate the degree of involvement of WD40 domains in multi-domain WD40 proteins.

### Gene expression analysis

Expression data of WD40 genes were obtained from the RNA-seq dataset in Human Protein Atlas database, which assayed the expression levels of coding RNAs from 95 individuals in 27 different human tissues^35^. The gene expression levels were denoted by FPKM (Fragments Per Kilobase of transcript per Million fragments mapped), and the data were downloaded from ArrayExpress website (ID: E-MTAB-1733). For each tissue, the FPKM values of every gene were averaged among all individual samples. Consistent with the original article^35^, genes with FPKM less than 1.0 in all 27 tissues were termed as “Not detected”, and were treated as 0 in the fold change calculation. Genes with FPKM greater than 1.0 in all 27 tissues were defined as “Expressed in all”, and if all are greater than 10, they were termed as “Highly expressed in all” or “House-keeping genes”. The “Tissue-specific” WD40 genes were defined as genes whose FPKM values in a specific tissue are 5 folds greater than in all other tissues, which includes “Tissue-specific” and “Tissue-enriched” in the original article.

## Additional Information

**How to cite this article**: Zou, X.-D. *et al*. Genome-wide Analysis of WD40 Protein Family in Human. *Sci. Rep.* **6**, 39262; doi: 10.1038/srep39262 (2016).

**Publisher's note:** Springer Nature remains neutral with regard to jurisdictional claims in published maps and institutional affiliations.

## Supplementary Material

Supplementary Information

Supplementary Dataset 1

## Figures and Tables

**Table 1:** The *hs*WD40 genes and the chromosomes they belong to.

Gene	Chr	Gene	Chr	Gene	Chr	Gene	Chr	Gene	Chr	Gene	Chr
AAAS	12	DCAF5	14	GNB3	12	POC1A	3	TEP1	14	WDR49	3
AAMP	2	DCAF7	17	GNB4	3	POC1B	12	THOC3	5	WDR53	3
AHI1	6	DCAF8	1	GNB5	15	PPP2R2A	8	THOC6	16	WDR54	2
APAF1	12	DCAF8L1	X	GTF3C2	2	PPP2R2B	5	TLE1	9	WDR55	5
ARPC1A	7	DCAF8L2	X	HERC1	15	PPP2R2C	4	TLE2	19	WDR5	9
ARPC1B	7	DDB1	11	HIRA	22	PPP2R2D	10	TLE3	15	WDR59	16
ATG16L1	2	DDB2	11	HPS5	11	PPWD1	5	TLE4	9	WDR5B	3
ATG16L2	11	DENND3	8	IFT122	3	PREB	2	TLE6	19	WDR60	7
BOP1	8	DMXL1	5	IFT140	16	PRPF19	11	TRAF7	16	WDR61	15
BRWD1	21	DMXL2	15	IFT172	2	PRPF4	9	TSSC1	2	WDR62	19
BRWD3	X	DNAI1	9	IFT80	3	PWP1	12	UTP15	5	WDR6	3
BTRC	10	DNAI2	17	KATNB1	16	PWP2	21	UTP18	17	WDR63	1
BUB3	10	DPH7	9	KCTD3	1	RAE1	20	VPRBP	3	WDR64	1
CDC20	1	DTL	1	KIAA1875	8	RBBP4	1	WDFY1	2	WDR66	12
CDC20B	5	DYNC1I1	7	KIF21A	12	RBBP5	1	WDFY2	13	WDR70	5
CDC40	6	DYNC1I2	2	KIF21B	1	RBBP7	X	WDHD1	14	WDR7	18
CDRT1	17	EDC4	16	LRBA	4	RFWD2	1	WDR12	2	WDR72	15
CFAP43	10	EED	11	LRWD1	7	RIC1	9	WDR13	X	WDR73	15
CFAP44	3	EIF2A	3	LYST	1	RPTOR	17	WDR1	4	WDR74	11
CFAP52	17	EIF3B	7	MAPKBP1	15	RRP9	3	WDR17	4	WDR75	2
CFAP57	1	EIF3I	1	MED16	19	SCAP	3	WDR18	19	WDR76	15
CHAF1B	21	ELP2	18	MIOS	7	SEC13	3	WDR19	4	WDR77	1
CIAO1	2	EML1	14	MLST8	16	SEC31A	4	WDR20	14	WDR78	1
CIRH1A	16	EML2	19	NBEA	13	SEC31B	10	WDR24	16	WDR81	17
COPA	1	EML3	11	NBEAL1	2	SEH1L	18	WDR25	14	WDR82	3
COPB2	3	EML4	2	NBEAL2	3	SHKBP1	19	WDR26	1	WDR83	19
CORO1A	16	EML5	14	NEDD1	12	SMU1	9	WDR27	6	WDR86	7
CORO1B	11	EML6	2	NLE1	17	SNRNP40	1	WDR3	1	WDR87	19
CORO1C	12	ERCC8	5	NOL10	2	SPAG16	2	WDR31	9	WDR88	19
CORO2A	9	FBXW10	17	NSMAF	8	STRAP	12	WDR33	2	WDR89	14
CORO2B	15	FBXW11	5	NUP37	12	STRN	2	WDR34	9	WDR90	16
CORO6	17	FBXW12	3	NUP43	6	STRN3	14	WDR35	2	WDR91	7
CORO7	16	FBXW2	9	NWD1	19	STRN4	19	WDR36	5	WDR92	2
CSTF1	20	FBXW4	10	NWD2	4	STXBP5	6	WDR37	10	WDSUB1	2
DAW1	2	FBXW5	9	PAAF1	11	STXBP5L	3	WDR38	9	WDTC1	1
DCAF10	9	FBXW7	4	PAFAH1B1	17	TAF5	10	WDR41	5	WIPI1	17
DCAF11	14	FBXW8	12	PAK1IP1	6	TAF5L	1	WDR4	21	WIPI2	7
DCAF12	9	FBXW9	19	PALB2	16	TBC1D31	8	WDR43	2	WRAP53	17
DCAF12L1	X	FZR1	19	PAN2	12	TBL1XR1	3	WDR44	X	WRAP73	1
DCAF12L2	X	GEMIN5	5	PEX7	6	TBL1X	X	WDR45B	17	WSB1	17
DCAF13	8	GNB1	1	PHIP	6	TBL1Y	Y	WDR45	X	WSB2	12
DCAF4	14	GNB1L	22	PIK3R4	3	TBL2	7	WDR46	6	ZNF106	15
DCAF4L1	4	GNB2	7	PLAA	9	TBL3	16	WDR47	1		
DCAF4L2	8	GNB2L1	5	PLRG1	4	TECPR2	14	WDR48	3		

**Table 2:** The “tissue-specific” *hs*WD40 genes.

Tissue	Gene symbol
Testis	CDC20B, DAW1, DCAF12L1, DCAF4L1, DCAF4L2, DCAF8L1, DCAF8L2, FBXW10, KIAA1875, LRWD1, TBL2, WDR62, WDR64, WDR65, WDR78, WDR87, WDR88
Brain	NWD2
Pancreas	FBXW12
Prostate	TBL1Y

## References

[b1] Stirnimann C. U., Petsalaki E., Russell R. B. & Muller C. W. WD40 proteins propel cellular networks. Trends Biochem. Sci. 35, 565–574 (2010). 20451393 10.1016/j.tibs.2010.04.003

[b2] Smith T. F., Gaitatzes C., Saxena K. & Neer E. J. The WD repeat: a common architecture for diverse functions. Trends Biochem. Sci. 24, 181–185 (1999). 10322433 10.1016/s0968-0004(99)01384-5

[b3] Li D. & Roberts R. WD-repeat proteins: structure characteristics, biological function, and their involvement in human diseases. Cell Mol. Life Sci. 58, 2085–2097 (2001). 11814058 10.1007/PL00000838 PMC11337334

[b4] Neer E. J., Schmidt C. J., Nambudripad R. & Smith T. F. The ancient regulatory-protein family of WD-repeat proteins. Nature 371, 297–300 (1994). 8090199 10.1038/371297a0

[b5] Xu C. & Min J. Structure and function of WD40 domain proteins. Protein Cell 2, 202–214 (2011). 21468892 10.1007/s13238-011-1018-1 PMC4875305

[b6] Gaudet R., Bohm A. & Sigler P. B. Crystal structure at 2.4 angstroms resolution of the complex of transducin betagamma and its regulator, phosducin. Cell 87, 577–588 (1996). 8898209 10.1016/s0092-8674(00)81376-8

[b7] Ruthenburg A. J. . Histone H3 recognition and presentation by the WDR5 module of the MLL1 complex. Nat. Struct. Mol. Biol. 13, 704–712 (2006). 16829959 10.1038/nsmb1119 PMC4698793

[b8] Wakasugi M. . DDB accumulates at DNA damage sites immediately after UV irradiation and directly stimulates nucleotide excision repair. J. Biol. Chem. 277, 1637–1640 (2002). 11705987 10.1074/jbc.C100610200

[b9] Jennings B. H., Pickles L. M., Wainwright S. M., Roe S. M., Pearl L. H. & Ish-Horowicz D. Molecular recognition of transcriptional repressor motifs by the WD domain of the Groucho/TLE corepressor. Mol. Cell 22, 645–655 (2006). 16762837 10.1016/j.molcel.2006.04.024

[b10] Znaidi S., Pelletier B., Mukai Y. & Labbe S. The Schizosaccharomyces pombe corepressor Tup11 interacts with the iron-responsive transcription factor Fep1. J. Biol. Chem. 279, 9462–9474 (2004). 14668334 10.1074/jbc.M312787200

[b11] Yan C., Hang J., Wan R., Huang M., Wong C. C. & Shi Y. Structure of a yeast spliceosome at 3.6-angstrom resolution. Science 349, 1182–1191 (2015). 26292707 10.1126/science.aac7629

[b12] Higa L. A., Wu M., Ye T., Kobayashi R., Sun H. & Zhang H. CUL4-DDB1 ubiquitin ligase interacts with multiple WD40-repeat proteins and regulates histone methylation. Nat. Cell Biol. 8, 1277–1283 (2006). 17041588 10.1038/ncb1490

[b13] Zou H., Henzel W. J., Liu X., Lutschg A. & Wang X. Apaf-1, a human protein homologous to C. elegans CED-4, participates in cytochrome c-dependent activation of caspase-3. Cell 90, 405–413 (1997). 9267021 10.1016/s0092-8674(00)80501-2

[b14] Zhan P. . FBXW7 negatively regulates ENO1 expression and function in colorectal cancer. Lab Invest. 95, 995–1004 (2015). 26097998 10.1038/labinvest.2015.71 PMC4552619

[b15] Wang X. . Fbxw7 regulates hepatocellular carcinoma migration and invasion via Notch1 signaling pathway. Int. J. Oncol. 47, 231–243 (2015). 25955618 10.3892/ijo.2015.2981

[b16] Ramasamy S. . Tle1 tumor suppressor negatively regulates inflammation *in vivo* and modulates NF-kappaB inflammatory pathway. Proc. Natl. Acad. Sci. USA 113, 1871–1876 (2016). 26831087 10.1073/pnas.1511380113 PMC4763742

[b17] Ozawa T. . A novel WDR45 mutation in a patient with static encephalopathy of childhood with neurodegeneration in adulthood (SENDA). Am. J. Med. Genet. A. 164A, 2388–2390 (2014). 25044655 10.1002/ajmg.a.36635 PMC4278411

[b18] Nicholas A. K. . WDR62 is associated with the spindle pole and is mutated in human microcephaly. Nat. Genet. 42, 1010–1014 (2010). 20890279 10.1038/ng.682 PMC5605390

[b19] van Nocker S. & Ludwig P. The WD-repeat protein superfamily in Arabidopsis: conservation and divergence in structure and function. BMC Genomics 4, 50 (2003). 14672542 10.1186/1471-2164-4-50 PMC317288

[b20] Ouyang Y., Huang X., Lu Z. & Yao J. Genomic survey, expression profile and co-expression network analysis of OsWD40 family in rice. BMC genomics 13, 100 (2012). 22429805 10.1186/1471-2164-13-100 PMC3329404

[b21] Li Q., Zhao P., Li J., Zhang C., Wang L. & Ren Z. Genome-wide analysis of the WD-repeat protein family in cucumber and Arabidopsis. Mol. Genet. Genomics 289, 103–124 (2014). 24292651 10.1007/s00438-013-0789-x

[b22] Mishra A. K., Muthamilarasan M., Khan Y., Parida S. K. & Prasad M. Genome-wide investigation and expression analyses of WD40 protein family in the model plant foxtail millet (Setaria italica L.). PLoS One 9, e86852 (2014). 24466268 10.1371/journal.pone.0086852 PMC3900672

[b23] Zhu Y. . Genome-wide identification, sequence characterization, and protein-protein interaction properties of DDB1 (damaged DNA binding protein-1)-binding WD40-repeat family members in Solanum lycopersicum. Planta 241, 1337–1350 (2015). 25680350 10.1007/s00425-015-2258-8

[b24] Wang Y., Jiang F., Zhuo Z., Wu X. H. & Wu Y. D. A method for WD40 repeat detection and secondary structure prediction. PLoS One 8, e65705 (2013). 23776530 10.1371/journal.pone.0065705 PMC3679165

[b25] Magrane M. & Consortium U. UniProt Knowledgebase: a hub of integrated protein data. Database (Oxford) 2011, bar009 (2011). 10.1093/database/bar009 PMC3070428 21447597

[b26] Zhang J. Evolution by gene duplication: an update. Trends Ecol. Evol. 18, 292–298 (2003).

[b27] Vogel C., Teichmann S. A. & Pereira-Leal J. The relationship between domain duplication and recombination. J. Mol. Biol. 346, 355–365 (2005). 15663950 10.1016/j.jmb.2004.11.050

[b28] Chaudhuri I., Soding J. & Lupas A. N. Evolution of the beta-propeller fold. Proteins 71, 795–803 (2008). 17979191 10.1002/prot.21764

[b29] Zhang L., Lu H. H., Chung W. Y., Yang J. & Li W. H. Patterns of segmental duplication in the human genome. Mol. Biol. Evol. 22, 135–141 (2005). 15371527 10.1093/molbev/msh262

[b30] Shoja V. & Zhang L. A roadmap of tandemly arrayed genes in the genomes of human, mouse, and rat. Mol. Biol. Evol. 23, 2134–2141 (2006). 16901985 10.1093/molbev/msl085

[b31] Collins F., Lander E., Rogers J., Waterston R. & Conso I. Finishing the euchromatic sequence of the human genome. Nature 431, 931–945 (2004). 15496913 10.1038/nature03001

[b32] Elsayed S. M. . Non-manifesting AHI1 truncations indicate localized loss-of-function tolerance in a severe Mendelian disease gene. Hum. Mol. Genet. 24, 2594–2603 (2015). 25616960 10.1093/hmg/ddv022 PMC4383865

[b33] Schaefer M. H., Fontaine J. F., Vinayagam A., Porras P., Wanker E. E. & Andrade-Navarro M. A. HIPPIE: Integrating protein interaction networks with experiment based quality scores. PLoS One 7, e31826 (2012). 22348130 10.1371/journal.pone.0031826 PMC3279424

[b34] Guimaraes K. S., Jothi R., Zotenko E. & Przytycka T. M. Predicting domain-domain interactions using a parsimony approach. Genome Biol. 7, R104 (2006). 17094802 10.1186/gb-2006-7-11-r104 PMC1794579

[b35] Fagerberg L. . Analysis of the human tissue-specific expression by genome-wide integration of transcriptomics and antibody-based proteomics. Mol. Cell Proteomics 13, 397–406 (2014). 24309898 10.1074/mcp.M113.035600 PMC3916642

[b36] Humphray S. J. . DNA sequence and analysis of human chromosome 9. Nature 429, 369–374 (2004). 15164053 10.1038/nature02465 PMC2734081

[b37] Saitsu H. . De novo mutations in the autophagy gene WDR45 cause static encephalopathy of childhood with neurodegeneration in adulthood. Nat. Genet. 45, 445–449, 449e441 (2013). 23435086 10.1038/ng.2562

[b38] Park J. Y. . Breast cancer-associated missense mutants of the PALB2 WD40 domain, which directly binds RAD51C, RAD51 and BRCA2, disrupt DNA repair. Oncogene 33, 4803–4812 (2014). 24141787 10.1038/onc.2013.421 PMC3994186

[b39] Park J. Y., Zhang F. & Andreassen P. R. PALB2: the hub of a network of tumor suppressors involved in DNA damage responses. BBA-Rev. Cancer 1846, 263–275 (2014). 10.1016/j.bbcan.2014.06.003 PMC4183126 24998779

[b40] Flicek P. . Ensembl 2013. Nuleic Acids Res. 41, D48–55 (2013). 10.1093/nar/gks1236 PMC3531136 23203987

[b41] Jones P. . InterProScan 5: genome-scale protein function classification. Bioinformatics 30, 1236–1240 (2014). 24451626 10.1093/bioinformatics/btu031 PMC3998142

[b42] Liu W. . IBS: an illustrator for the presentation and visualization of biological sequences. Bioinformatics 31, 3359–3361 (2015). 26069263 10.1093/bioinformatics/btv362 PMC4595897

[b43] Huang da W., Sherman B. T. & Lempicki R. A. Systematic and integrative analysis of large gene lists using DAVID bioinformatics resources. Nat. Protoc. 4, 44–57 (2009). 19131956 10.1038/nprot.2008.211

[b44] Huang da W., Sherman B. T. & Lempicki R. A. Bioinformatics enrichment tools: paths toward the comprehensive functional analysis of large gene lists. Nucleic Acids Res. 37, 1–13 (2009). 19033363 10.1093/nar/gkn923 PMC2615629

[b45] Camacho C. . BLAST+: architecture and applications. BMC Bioinformatics 10, 421 (2009). 20003500 10.1186/1471-2105-10-421 PMC2803857

[b46] Shannon P. . Cytoscape: a software environment for integrated models of biomolecular interaction networks. Genome Res. 13, 2498–2504 (2003). 14597658 10.1101/gr.1239303 PMC403769

[b47] Krzywinski M. . Circos: an information aesthetic for comparative genomics. Genome Res. 19, 1639–1645 (2009). 19541911 10.1101/gr.092759.109 PMC2752132

[b48] Pan D. & Zhang L. Tandemly arrayed genes in vertebrate genomes. Comp. Funct. Genomics. 545269 (2008). 10.1155/2008/545269 PMC2547482 18815629

[b49] Sonnhammer E. L. & Ostlund G. InParanoid 8: orthology analysis between 273 proteomes, mostly eukaryotic. Nuleic Acids Res. 43, D234–239 (2015). 10.1093/nar/gku1203 PMC4383983 25429972

