# Supplementary material for: Genome-wide Analysis of WD40 Protein Family in Human
Source: Sci Rep. 2016 Dec 19;6:39262. doi: 10.1038/srep39262 (PMC5172248; doi:10.1038/srep39262)
Supplement: Supplementary Information [file srep39262-s1.pdf]

Supplementary Information for "Genome-wide Analysis of WD40 Protein Family in Human"

Xu-Dong Zou[1], Xue-Jia Hu[1], Jing Ma[1], Tuan Li[1], Zhi-Qiang Ye[1,*] & Yun-Dong Wu[1,2]

[1] Lab of Computational Chemistry and Drug Design, Laboratory of Chemical Genomics, Peking University Shenzhen Graduate School, Shenzhen 518055, P. R. China

[2] College of Chemistry, Peking University, Beijing, 100871, P. R. China

Supplementary Figures

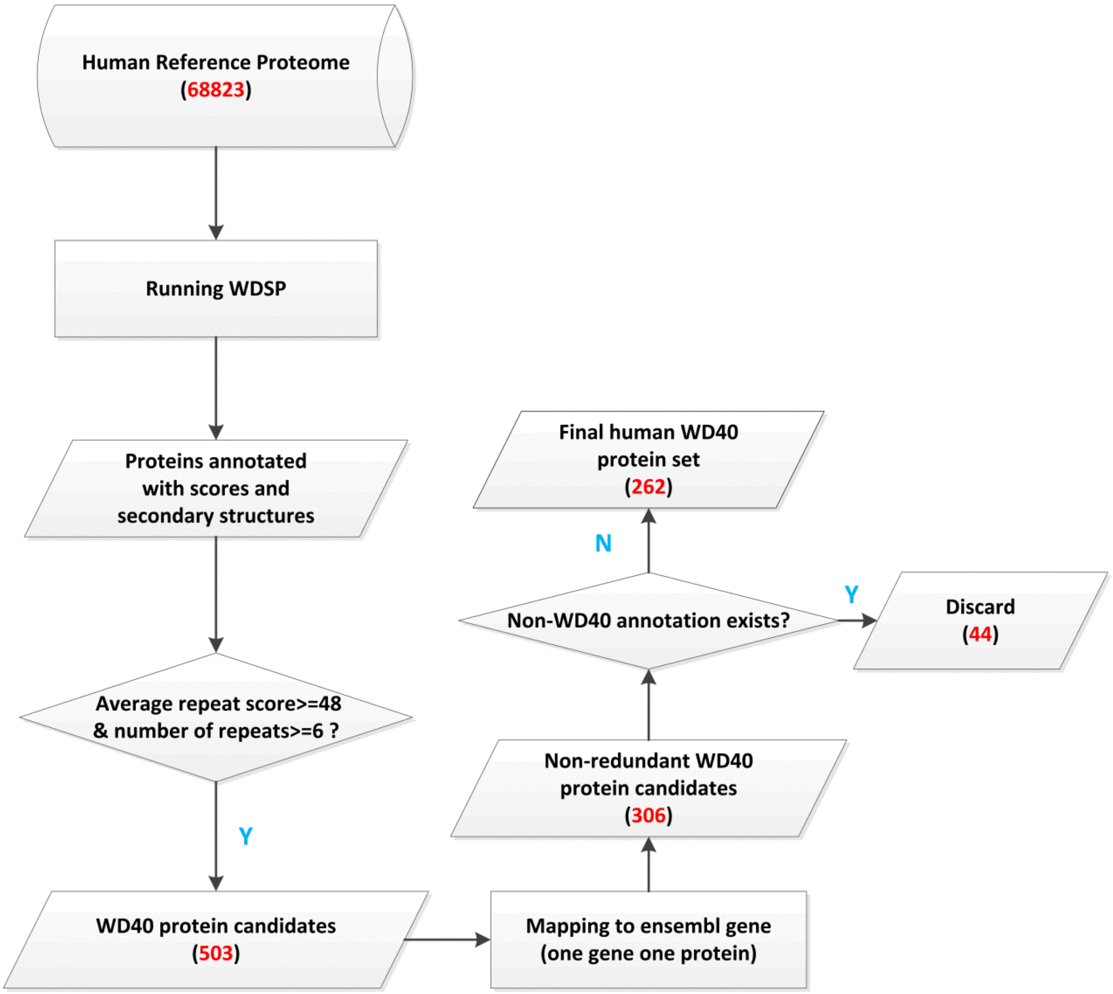

Supplementary Figure S1. Flowchart of the identification of *hs*WD40 genes.

The proteins in the human reference proteome were analysed by WDSP, and the

filtering score was set to 48 for the average score of repeats, and only proteins with 6 or more repeats were considered as WD40 proteins. The WD40 protein candidates were mapped to Ensembl genes for screening the longest one for each gene. The red numbers in the parentheses give the counts of proteins left after the corresponding steps.

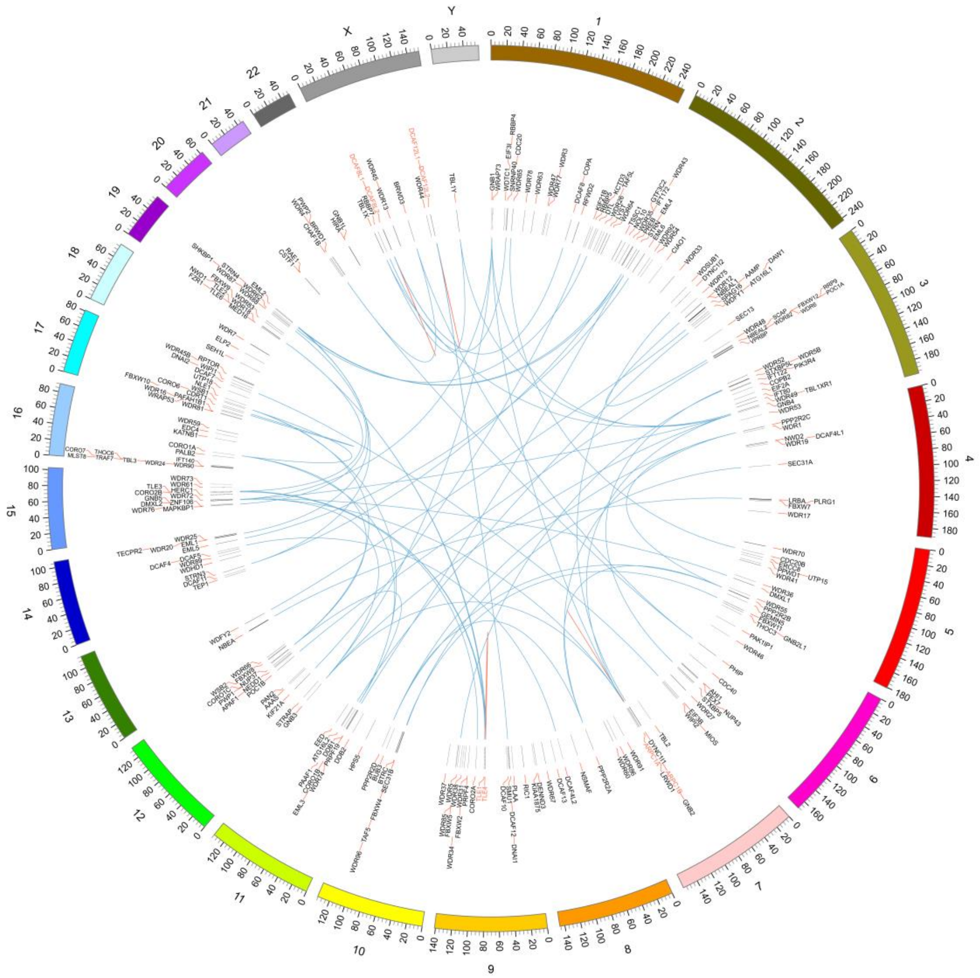

Supplementary Figure S2. The human “WD40 map”.

The *hs*WD40 genes were plotted according to their chromosomal locations. From the outside to the inside, the first track delineates 24 chromosomes in different colours,

and the second track presents the symbols of the *hs*WD40 genes, whose positions are denoted at the third track with black short lines. The innermost blue curves connect the highly similar domain pairs, except that pairs from the same gene were not shown. The four pairs of tandemly arrayed genes (TAGs) are marked in red.

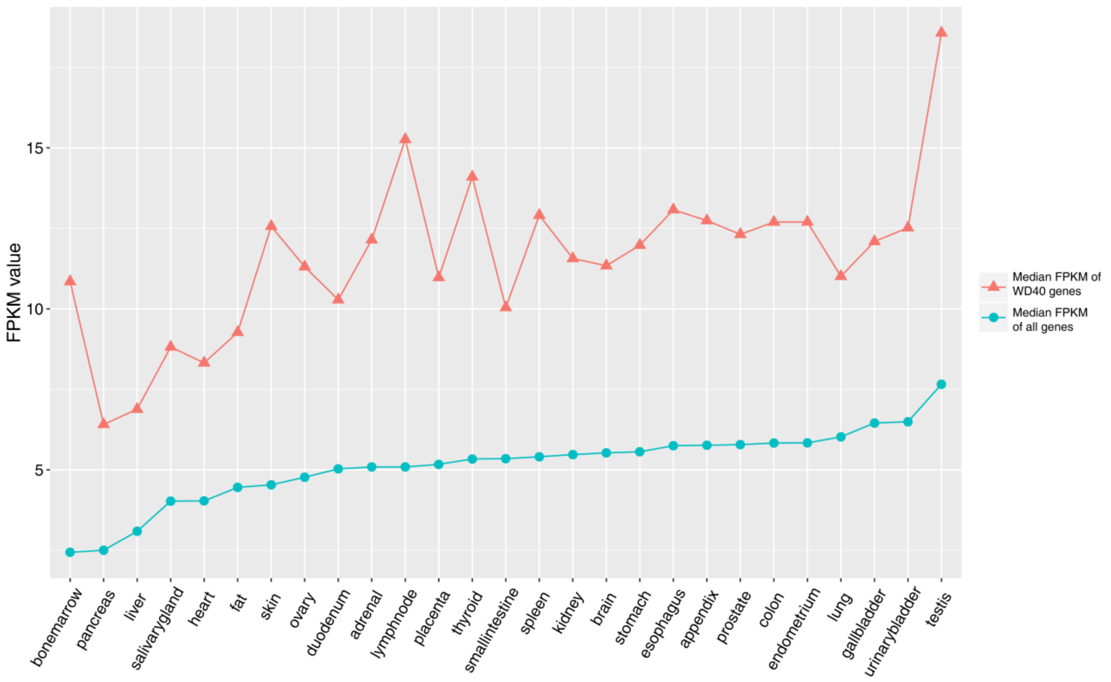

Supplementary Figure S3. Expression levels of *hs*WD40 genes and all genes in different human tissues.

The vertical axis denotes the gene expression levels in units of FPKM, while the horizontal axis gives the 27 human tissues. The triangles filled in red and the circles filled in cyan represent the median FPKM values of *hs*WD40 genes and all human genes, respectively.

Supplementary Tables

The data of supplementary table S1, S4, S5, S7, and S9 are provided in a spread sheet file online.

Supplementary Table S1. The full list of *hs*WD40 proteins with comprehensive information involved in this work.

The Ensembl gene ID, the Uniprot accession number and ID, and gene symbol are shown. The genomic location with strand orientation, the protein sequence length, the predicted WD40 repeat number, and the domain architecture were also provided. The numbers in the domain architecture indicate the boundaries of the domains in the full-length protein sequence.

Supplementary Table S2. Function annotations for proteins in each domain architecture.

*Class	Domain Architecture	Functions or biological roles
Class 2	F-box + WD40	Substrates receptor in SCF ubiquitin ligase system[1]
Class 3	LisH + WD40	Protein dimerization; histone binding; transcription repression[2, 3]
Class 4	[PH-BEACH/GRAM] + BEACH + WD40	Affecting lysosome size (LYST, NSMAF), apoptosis (NSMAF), autophagy (LYST, LRBA) or synapse formation[4]
Class 5	HELP + WD40	Microtubule destabilization[5] and microtubule formation[6]
Class 6	WD40 + Utp	Component of UTPB complex and involved in rRNA processing[7]
Class 7	TLE_N + WD40	Co-repression of transcription by binding with transcription factors[8]
Class 8	WD40 + Bromodomain	Histone acetylation reader[9]; regulation of cell morphology and cytoskeletal organization[10]
Class 9	Striatin N-terminal + WD40	Calmodulin binding[11]; Estrogen Receptor binding[12]

Class 10	NLE + WD40	Component of PeBoW complex and involved in rRNA processing and ribosomal biogenesis[13]; modulate NOTCH signaling pathway[14]
Class 11	NACHT + WD40	Component of telomerase[15]; modulator of androgen receptor signaling[16]
Class 12	BTB/POZ-like + WD40	Substrates adaptors of CUL3 ubiquitin ligase[17]; EGFR signaling[18]
Class 13	ATG16 + WD40	Involved in autophagy[19, 20]
Class 14	Dynein_IC2 + WD40	Component of dynein 1 complex; microtubule motor activity[21]
Class 15	RING finger + WD40	Zinc ion binding; E3 ubiquitin ligase[22]
Class 16	WD40 + Lgl_C	Syntaxin binding; Neurotransmitter release process[23]; regulate cell polarization[24]
Class 17	Kinesin motor + WD40	Microtubule motor activity[25]
Class 18	TFIID_90kDa + WD40	Subunit of TFIID complex[26] and PCAF histone acetylation complex[27]
Class 19	WD40 + SOCS box	E3 ubiquitin protein ligase[28]
Class 20	Rav1p_C + WD40	Regulate exocytosis of neurotransmitter[29]

[*] Class 1 and 21 are not included in this table, since Class 1 consists of proteins with only WD40 domains and Class 21 includes many different domain architectures together.

Supplementary Table S3. Enriched GO terms of biological process for genes belonging to animal-specific domain architectures.

GO ID	GO terms	Genes	p*
GO:1904837	beta-catenin-TCF complex assembly	TLE1, TLE2, TLE3, TLE4	1.7E-5
GO:0016055	Wnt signaling pathway	STRN, TLE1, TLE2, TLE3, TLE4	6.3E-5
GO:0007018	microtubule-based movement	DYNC1I1, DYNC1I2, KIF21A, KIF21B	1.2E-4
GO:0000226	microtubule cytoskeleton organization	EML1, EML3, EML4	3.2E-3
GO:0009887	animal organ morphogenesis	TLE1, TLE2, TLE3	5.3E-3
GO:0051260	protein homooligomerization	APAF1, KCTD3, SHKBP1	1.9E-2

* p is the *p*-value and its cutoff for significance was set to less than 0.05.

The GO enrichment was consulted on 26 WD40 proteins with animal-specific domain architectures by DAVID.

Supplementary Table S4. The highly similar WD40 domain pairs identified by the BLASTP program.

Supplementary Table S5. The highly similar WD40 repeat pairs identified by the BLASTP program.

Supplementary Table S6. The counts of *hs*WD40 genes and all protein-coding genes in each chromosome.

Chr	# of Protein-coding genes	# of WD40 genes	Percentage of WD40 genes	P-value
1	2,076	24	1.156%	0.328
2	1,281	24	1.874%	0.043
3	1,078	22	2.041%	0.023
4	767	10	1.304%	0.531
5	893	14	1.568%	0.264
6	1,052	9	0.856%	0.123
7	917	12	1.309%	0.521
8	701	8	1.141%	0.447
9	805	17	2.112%	0.032
10	771	8	1.038%	0.333
11	1,317	10	0.759%	0.043
12	1,070	14	1.308%	0.517
13	329	2	0.608%	0.200

14	652	12	1.840%	0.139
15	617	11	1.783%	0.175
16	875	14	1.600%	0.241
17	1,208	15	1.242%	0.507
18	288	3	1.042%	0.489
19	1,481	14	0.945%	0.133
20	560	2	0.357%	0.023
21	242	4	1.653%	0.380
22	450	2	0.444%	0.068
X	830	10	1.205%	0.493
Y	54	1	1.852%	0.504

The numbers of protein-coding genes were retrieved from Ensembl Human Genome GRCh37.p13 (Ensembl 75, data frozen in March, 2014), and there are 20,314 protein-coding genes in 24 chromosomes. The overall percentage of WD40 genes in all protein-coding genes is 1.290% (262 divided by 20,314). For those chromosomes whose percentages of WD40 genes are greater than 1.290%, the numbers were shaded in red. P-values were calculated based on hyper-geometric distributions. For those percentages less than (greater than) 1.29%, the lower-tail (upper-tail) setting was enabled. The p-values less than 0.05 were shaded in yellow.

Supplementary Table S7. The orthlogs of human WD40 proteins in Drosophila, Arabidopsis, and yeast.

Supplementary Table S8. The counts and percentages of *hs*WD40 genes and all human genes with different phylogenetic patterns.

Drosophila	Arabidopsis	Yeast	# of *hs*WD40 genes	# of all human genes
+	+	+	70 (26.72%)	2,349 (11.27%)

+	+	-	45 (17.18%)	1,716 (8.24%)
-	+	+	5 (1.91%)	533 (2.56%)
+	-	+	13 (4.96%)	540 (2.59%)
-	+	-	18 (6.87%)	972 (4.67%)
+	-	-	54 (20.61%)	3,985 (19.13%)
-	-	+	3 (1.15%)	251 (1.20%)
-	-	-	54 (20.61%)	10,488 (50.34%)
**Total**			**262 (100%)**	**20,834 (100%)**

The phylogenetic patterns are defined by the ortholog existence statuses in Drosophila, Arabidopsis, and yeast. The '+' denotes that there exist orthologs of human genes in the corresponding species, while the '-' denotes that no ortholog was found in the corresponding species. Column "# of *hs*WD40 genes" shows the number of *hs*WD40 genes with a corresponding phylogenetic pattern, while column "# of all human genes" shows the number of all human genes accordingly. The percentages were calculated by dividing the numbers by the total number of *hs*WD40 genes (262) and the total number of all human genes (20,834 according to the corresponding version of Inparanoid database), respectively.

Supplementary Table S9. The expression profiles of *hs*WD40 genes across 27 different human tissues.

The expression levels are denoted in units of FPKM, and the colour schema are explained in the bottom of the table.

References

1. Xu C, Min J. Structure and function of WD40 domain proteins. *Protein Cell* **2**, 202-214 (2011).

2. Gerlitz G, Darhin E, Giorgio G, Franco B, Reiner O. Novel functional features of the Lis-H domain: role in protein dimerization, half-life and cellular localization. *Cell Cycle* **4**,

1632-1640 (2005).

3. Choi HK, *et al.* Function of multiple Lis-Homology domain/WD-40 repeat-containing proteins in feed-forward transcriptional repression by silencing mediator for retinoic and thyroid receptor/nuclear receptor corepressor complexes. *Mol Endocrinol* **22**, 1093-1104 (2008).

4. Cullinane AR, Schaffer AA, Huizing M. The BEACH is hot: a LYST of emerging roles for BEACH-domain containing proteins in human disease. *Traffic* **14**, 749-766 (2013).

5. Eichenmuller B, Everley P, Palange J, Lepley D, Suprenant KA. The human EMAP-like protein-70 (ELP70) is a microtubule destabilizer that localizes to the mitotic apparatus. *The Journal of biological chemistry* **277**, 1301-1309 (2002).

6. Pollmann M, Parwaresch R, Adam-Klages S, Kruse ML, Buck F, Heidebrecht HJ. Human EML4, a novel member of the EMAP family, is essential for microtubule formation. *Experimental cell research* **312**, 3241-3251 (2006).

7. Zhang C, Lin J, Liu W, Chen X, Chen R, Ye K. Structure of Utp21 tandem WD domain provides insight into the organization of the UTPB complex involved in ribosome synthesis. *PLoS One* **9**, e86540 (2014).

8. Jennings BH, Ish-Horowicz D. The Groucho/TLE/Grg family of transcriptional co-repressors. *Genome Biol* **9**, 205 (2008).

9. Filippakopoulos P, *et al.* Histone recognition and large-scale structural analysis of the human bromodomain family. *Cell* **149**, 214-231 (2012).

10. Bai SW, *et al.* Identification and characterization of a set of conserved and new regulators of cytoskeletal organization, cell morphology and migration. *BMC biology* **9**, 54 (2011).

11. Castets F, Rakitina T, Gaillard S, Moqrich A, Mattei MG, Monneron A. Zinedin, SG2NA, and striatin are calmodulin-binding, WD repeat proteins principally expressed in the brain. *The Journal of biological chemistry* **275**, 19970-19977 (2000).

12. Lu Q, Pallas DC, Surks HK, Baur WE, Mendelsohn ME, Karas RH. Striatin assembles a membrane signaling complex necessary for rapid, nongenomic activation of endothelial NO synthase by estrogen receptor alpha. *Proc Natl Acad Sci U S A* **101**, 17126-17131 (2004).

13. Holzel M, *et al.* Mammalian WDR12 is a novel member of the Pes1-Bop1 complex and is required for ribosome biogenesis and cell proliferation. *The Journal of cell biology* **170**, 367-378 (2005).

14. Royet J, Bouwmeester T, Cohen SM. Notchless encodes a novel WD40-repeat-containing

protein that modulates Notch signaling activity. *Embo J* **17**, 7351-7360 (1998).

15. Saito T, *et al.* Comparative gene mapping of the human and mouse TEP1 genes, which encode one protein component of telomerases. *Genomics* **46**, 46-50 (1997).

16. Correa RG, Krajewska M, Ware CF, Gerlic M, Reed JC. The NLR-related protein NWD1 is associated with prostate cancer and modulates androgen receptor signaling. *Oncotarget* **5**, 1666-1682 (2014).

17. Geyer R, Wee S, Anderson S, Yates J, Wolf DA. BTB/POZ domain proteins are putative substrate adaptors for cullin 3 ubiquitin ligases. *Molecular cell* **12**, 783-790 (2003).

18. Feng L, Wang JT, Jin H, Qian K, Geng JG. SH3KBP1-binding protein 1 prevents epidermal growth factor receptor degradation by the interruption of c-Cbl-CIN85 complex. *Cell biochemistry and function* **29**, 589-596 (2011).

19. Boada-Romero E, Letek M, Fleischer A, Pallauf K, Ramon-Barros C, Pimentel-Muinos FX. TMEM59 defines a novel ATG16L1-binding motif that promotes local activation of LC3. *Embo J* **32**, 566-582 (2013).

20. Dooley HC, Razi M, Polson HE, Girardin SE, Wilson MI, Tooze SA. WIPI2 links LC3 conjugation with PI3P, autophagosome formation, and pathogen clearance by recruiting Atg12-5-16L1. *Molecular cell* **55**, 238-252 (2014).

21. Asante D, Stevenson NL, Stephens DJ. Subunit composition of the human cytoplasmic dynein-2 complex. *Journal of cell science* **127**, 4774-4787 (2014).

22. Chasapis CT, Spyroulias GA. RING finger E(3) ubiquitin ligases: structure and drug discovery. *Current pharmaceutical design* **15**, 3716-3731 (2009).

23. Fujita Y, *et al.* Tomosyn: a syntaxin-1-binding protein that forms a novel complex in the neurotransmitter release process. *Neuron* **20**, 905-915 (1998).

24. Assemat E, Bazellieres E, Pallesi-Pocachard E, Le Bivic A, Massey-Harroche D. Polarity complex proteins. *Biochimica et biophysica acta* **1778**, 614-630 (2008).

25. Hirokawa N, Noda Y, Tanaka Y, Niwa S. Kinesin superfamily motor proteins and intracellular transport. *Nature reviews Molecular cell biology* **10**, 682-696 (2009).

26. Bieniossek C, *et al.* The architecture of human general transcription factor TFIID core complex. *Nature* **493**, 699-702 (2013).

27. Ogryzko VV, *et al.* Histone-like TAFs within the PCAF histone acetylase complex. *Cell* **94**, 35-44 (1998).

28. Kile BT, Schulman BA, Alexander WS, Nicola NA, Martin HM, Hilton DJ. The SOCS box: a tale of destruction and degradation. *Trends in biochemical sciences* **27**, 235-241 (2002).

29. Nagano F, *et al.* Rabconnectin-3, a novel protein that binds both GDP/GTP exchange protein and GTPase-activating protein for Rab3 small G protein family. *The Journal of biological chemistry* **277**, 9629-9632 (2002).
